# Rectal prolapse as a manifestation of inflammatory bowel disease with celiac disease in a 2-year-old male: a rare case report

**DOI:** 10.1097/MS9.0000000000000494

**Published:** 2023-04-06

**Authors:** Nafiza Martini, Nour Kara Tahhan, Mohamad S. Aldarwish, Jaber Mahmoud

**Affiliations:** a Faculty of Medicine, Damascus University; b Stemosis for Scientific Research; c Pediatric University Hospital, Damascus University, Damascus, Syrian Arab Republic

**Keywords:** case report, celiac disease, inflammatory bowel disease, rectal prolapse

## Abstract

The co-occurrence of inflammatory bowel disease (IBD) and celiac disease (CeD) is uncommon. The typical sign of this co-occurrence is malabsorption which leads to anemia, diarrhea, and malnutrition. In rare cases, recurrent rectal prolapse may also occur.

**Case presentation:**

A 2-year-old Syrian male baby presented with failure to thrive and chronic diarrhea for 18 months, along with recurrent rectal prolapse for the last 6 months. Biopsies taken confirmed a diagnosis of stage 3b celiac disease according to the Marsh classification. Furthermore, biopsies taken confirmed a diagnosis of IBD. Then, a high-fiber diet to manage IBD and celiac diet were both needed simultaneously, with signs of rectal prolapse, diarrhea, and bloating, occurring when either or both diets were stopped.

**Clinical discussion:**

The diagnosis was initially explained by the malnutrition and anemia. Even after the gluten-free diet, the patient showed no improvement in diarrhea and developed inferior gastrointestinal bleeding suggested anal fissure, infectious colitis, polyps, IBD, or solitary rectal ulcer syndrome. The relationship between celiac disease and IBD, in children, is still unclear. Current studies suggest that such co-occurrence is associated with higher risks of developing other autoimmune-related disorders, growth and puberty delay, and comorbidities.

**Conclusions:**

In the cases of pediatric co-occurrence of IBD and celiac disease, a conservative therapy consisting of two-fold diets for the two diseases should be tried first. If this step succeeds in controlling the clinical picture, it removes the necessity of introducing immunological pharmacologic treatments that may induce unfavorable side effects in a child.

## Introduction

Highlights

- The co-occurrence of inflammatory bowel disease (IBD), celiac disease, and recurrent rectal prolapse is uncommon.
- A 2-year-old Syrian male infant presented with failure to thrive and chronic diarrhea for 18 months, along with recurrent rectal prolapse for the last 6 months.
- A conservative therapy consisting of two-fold diets for the two diseases may remove the necessity of introducing immunological pharmacologic treatments that may induce unfavorable side effects in a pediatric patient.

Celiac disease is an immune disorder characterized by the destruction of the intestinal mucosa triggered by exposure to gluten in specific genetically predisposed individuals. Celiac disease patients complain of osmotic diarrhea, nonbloody diarrhea, steatorrhea, flatulence, and abdominal bloating. According to the last guidelines for the diagnosis of celiac disease testing of celiac-specific antibodies is the first-line test in high-risk patients, followed by duodenal biopsy in cases of positive serology or persistent suspicion of malabsorption, the histological findings reveal intraepithelial lymphocytic infiltration, crypt hyperplasia, and villous atrophy^1^.

IBD includes a group of inflammatory disorders containing Crohn’s disease and ulcerative colitis (UC)^2^. Crohn’s disease is a chronic inflammation that involves all mucosal layers of the intestinal wall. The inflammation occurs in discontinuous segments of the intestine (skip areas) and can lead to bowel stricture or fistulization. The main symptoms are weight loss, nonbloody diarrhea, and stomach aches.

In contrast, intestinal inflammation in UC is limited to the mucosa and submucosa, and the main symptoms are fecal urgency, bloody diarrhea, and abdominal pain. Children with UC co-occurring with associated autoimmune diseases most often display a less severe colonic disease course than patients with isolated UC^3^. Meanwhile, IBD has been associated with various autoimmune disorders, including systemic lupus erythematosus, primary sclerosing cholangitis, and celiac disease.

A diagnosis of IBD is suspected by medical history, and characteristic findings of physical examinations, and established by typical findings of imaging examinations such as endoscopy. colonoscopy should be conducted to establish a definite diagnosis of UC.

Upper gastrointestinal (GI) endoscopy had better be performed when a diagnosis of celiac disease is suspected^4^.

IBD prevalence in celiac disease is usually more severe in pediatric patients^5^, yet the incidence of recurrent rectal prolapse is still uncommon. Furthermore, rectal prolapse is more common among IBD patients than those with celiac disease^6^.

IBD is treated with anti-inflammatory medications, including 5-aminosalicylic acid derivatives, corticosteroids, and immunomodulators, while celiac disease is treated with dietary elimination of gluten through a lifelong gluten-free diet^7^. Herein, we present a case of IBD associated with celiac disease and rectal prolapse in a 2-year-old male child. The work has been reported in line with the SCARE 2020 criteria^8^

## Case presentation

**Figure 1:**
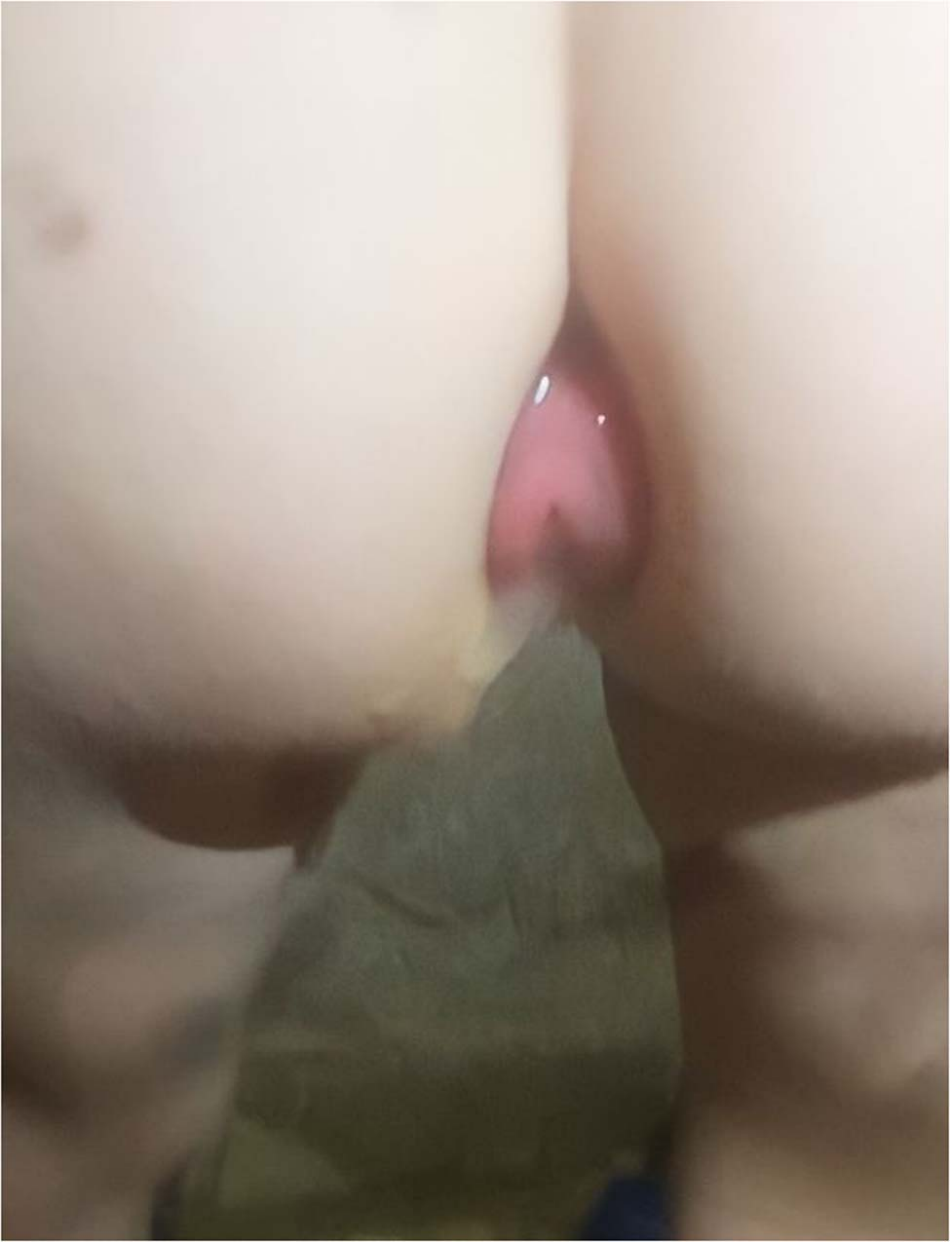
An image showing a part of the rectum protruding from the anus (rectal prolapse).

**Figure 2:**
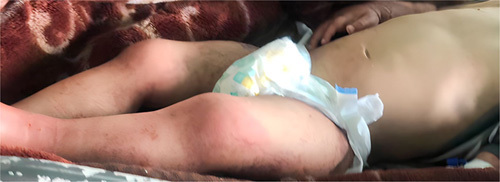
Skin changes in the context of severe acute malnutrition.

A 2-year-old Syrian male baby was admitted to the pediatric hospital. He presented with failure to thrive and chronic diarrhea for 18 months, along with recurrent rectal prolapse for the last 6 months. The frequency of rectal prolapse was once a week on average (Fig. 1) and the frequency of diarrhea was ten times a day at the time of the introduction of complementary foods at the age of 6 months. The diarrhea was often watery, profuse, nonmucous, and nonbloody. The baby had a normal vaginal delivery with normal birth weight. He has two sisters and six brothers, and there is no family history for such cases, no history of infectious disease and the baby takes no medications. He was exclusively breastfed for the first 4 months, after which he started receiving mixed feeding, then complementary food was added at 6 months of age. He presented to different healthcare units multiple times with no signs of clinical improvement. Both ceasing maternal breastfeeding and switching milk formulas had no effect. The baby weighed 3 kg at birth. He was 8.5 kg at 6 months and 8.9 at 1 year old. Presenting at the age of 2, he weighed only 9 kg. His height, head circumference, and mid-upper arm circumference were 85, 45, and 12 cm, respectively. Upon physical examination, the baby was moderately active. He had pronounced pallor and pitting edema of the face and lower extremities. His skin was dry, and there were scaly areas on the mouth, commissures, abdomen, and trunk, but there was no erythema or diaper dermatitis. A prolapse of the rectal mucosa through the anal orifice was observed and was subsequently retracted manually. Motor development was normal but with a weak reaction, lethargy, and general weakness. Due to the presence of growth failure, persistent diarrhea, rectal prolapse, and accompanying skin changes, the baby was diagnosed with severe acute malnutrition (Fig. 2).

The results of laboratory tests were as follows in Table 1.

**Table 1:** Laboratory tests

Test	Observed value	Reference range
White blood cells (µl)	11 600	4500-11 000
Neutrophils (%)	52	40–60
Lymphocytes (%)	43	20–40
Hemoglobin (g/dl)	5.9	9.5-13
Mean corpuscular volume (fl)	61	80–95
Platelets (µl)	437	150–450
Total protein (g/dl)	3.9	6–8.3
Albumin (g/dl)	1.9	3.2–4.8
Liver functions	Normal	—
Kidney functions	Normal	—
Protein to creatinine ratio	Normal	—

After intravenous blood and albumin transfusion, albumin, total protein, and hemoglobin values improved as follows: albumin 2.9 g/dl, total protein 4.2 g/dl, and hemoglobin10 g/dl.

Peripheral blood smear showed 2+ hypochromia and +1 anisocytosis leaning toward microcytosis. Otherwise, there was no evidence of spherocytosis or other abnormal morphologies, and white blood cells appeared normal.

The baby was put on a nothing-by-mouth diet for 24 h with intravenous fluids while monitoring diarrhea, which persisted. In a stool test, the laboratory results were as follows in Table 2.

**Table 2:** Stool analysis results

Lipids	Occult blood	White blood cells	Red blood cells	Reducing substances	pH
++++	Negative	5–10	Negative	Negative	7

**Figure 3:**
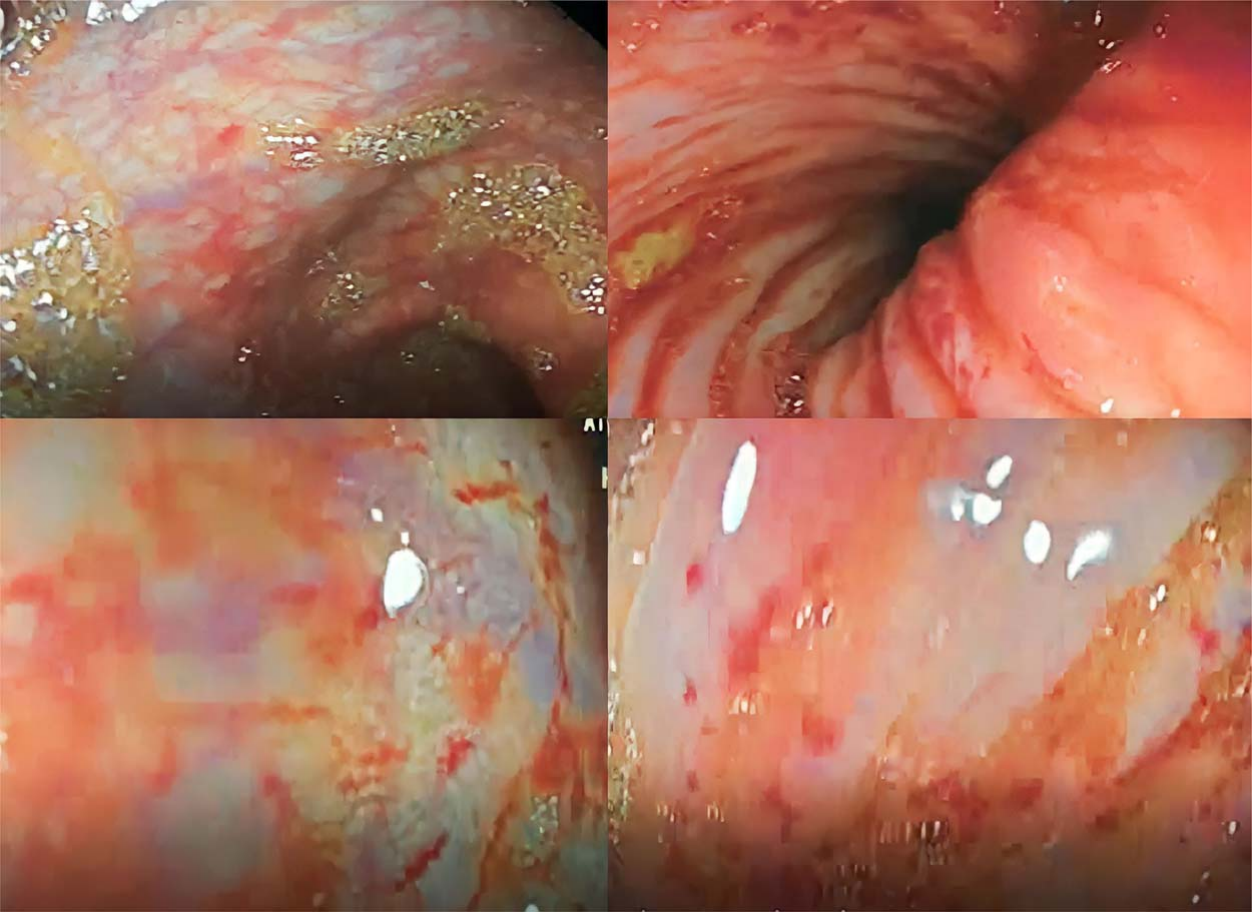
Lower gastrointestinal endoscopy showing punctate erosions less than 0.5 cm in the sigmoid and the rectum with redness of the mucous membrane.

These results suggested the diarrhea was secretory because diarrhea persisted despite the absolute diet of food during the first 24 h which means it was not osmotic diarrhea but secretory which in turn drew the presence of inflammation in the mucous. Many differential diseases were put in mind such as Eosinophilic gastroenteritis, fibrosis cystic, intestinal infections as well as celiac disease. Stool culture, urine test, abdominal ultrasound, arterial blood gases, sweat electrolytes, triglycerides, cholesterol, serum sodium, and serum chloride were all normal. Meanwhile, zinc and iron were low, and binding capacity was high. Furthermore, immunochemistry tests revealed an elevated anti-DGP IgA at 75 U/ml, supporting a diagnosis of celiac disease^9^. Biopsies were taken during an upper GI endoscopy and then sent to the pathology laboratory. The stomach showed preserved mucosa, no inflammatory infiltrates, and a negative Giemsa stain for *Helicobacter pylori*. The duodenum showed atrophic villi, one-third of their normal height. The crypts were hyperplastic. There was an increased count of interepithelial lymphocytes. Periodic acid–Schiff stain for specific pathogens was negative. Based on these results, a diagnosis of stage 3b celiac disease was confirmed according to the Marsh classification^10^. The baby was put on a diet of free-lactose milk at the beginning due to the possibility of developing secondary lactose intolerance since babies with IBD have damaged villi and lactase deficiency. This diet was later adjusted to a gluten-free one, comprising alternative starches such as corn, soybeans, and rice, and supplements of iron, folic acid, calcium, and magnesium, as needed. The patient showed initial improvement, with weight increasing to 9.2 kg and height to 85 cm, while head circumference and mid-upper arm circumference remained at 45 and 12 cm, respectively. However, he was readmitted 1 month later due to persisting diarrhea and rectal prolapse. Furthermore, he also presented with lower GI bleeding. He then weighed 9.3 kg. In a stool test, white blood cells and red blood cells were markedly positive, but the culture was negative. A lower GI endoscopy was subsequently performed, and it showed punctate erosions less than 0.5 cm in the sigmoid and the rectum with redness of the mucous membrane (Fig. 3). Several biopsies were taken from these erosions and then sent to the pathology laboratory. Colon biopsies showed an inflammatory infiltrate within the lamina propria containing some polymorphonuclear neutrophils, some of which were attacking glands with attempts to form diverticular abscesses. Some laboratory tests were performed as well (fecal calprotectin 200 μg/g, C-reactive protein was normal, erythrocyte sedimentation rate was 30 mm/h). Based on the histopathological result, a diagnosis of IBD-unclassified was confirmed and since the very early onset of the disease, monogenic IBD is suspected but because of the bad economic situation in Syria due to the war, we cannot afford genetic tests to validate this diagnosis. Therefore, a conservative course of management for IBD was chosen, consisting mainly of putting the baby on a high-fiber diet including fruits and vegetables in addition to the celiac disease diet. In a follow-up after 2 months, the baby weighed 10 kg, did not have diarrhea, and experienced a rectal prolapse only once. In a follow-up after 3 months, occult blood was positive in a stool test, but hemoglobin was 11 g/dl. The baby weighed 10.8 kg and showed no signs of either diarrhea or rectal prolapse. In a follow-up after 4 months, tissue transglutaminase IgA and fecal calprotectin tests were 16 U/ml and 47 μg/mg, respectively. The baby weighed 12.7 kg, had no signs of rectal prolapse, and suffered from diarrhea and bloating only if the diet was discontinued.

## Discussion

IBD prevalence in celiac disease patients is estimated at 1–3%, and the percentage is less in children.

The first reported co-occurrence case of IBD and celiac disease in a child in Syria was in 2014. To our knowledge, this was the only reported case, and it described a child having both celiac disease and Crohn’s disease. According to previous studies, the co-occurrence of celiac disease and UC is even much rarer. Furthermore, most cases previously reported included either a child over the age of 12 or an adult, and it is unusual to discover such co-occurrence in a child at such an early age as 2^11^.

The patient we presented here was diagnosed with celiac disease at the age of 2 after presenting with chronic severe diarrhea and recurrent rectal prolapse. The diagnosis was initially explained by the malnutrition and anemia the patient suffered from. Even after being put on a gluten-free diet, the patient showed no improvement in diarrhea and developed inferior GI bleeding. Mild lower GI bleeding in a child suggested anal fissure, infectious colitis, polyps, IBD, or solitary rectal ulcer syndrome.

The relationship between celiac disease and IBD, characteristics, and comorbidities in children is still unclear. Current studies suggest that such co-occurrence is associated with higher risks of developing other autoimmune-related disorders, growth and puberty delay, and comorbidities^12^.

Patients with celiac disease have an increased risk of developing IBDs. This refers to the possible genetic association between the two conditions and the immune-related characteristics^13^.

There is a suggested overlap between these two diseases, including genetic and environmental triggers but this remains understudied^14^.

The studied contribution of genetic predisposition is higher in celiac disease due to the HLA which is responsible for 40% of the genetic variance.

In contrast, only 14% of Crohn’s genetic risk alleles and 7.5% of UCs is known^15^.

Unresponsive malabsorption and anemia in celiac-diagnosed patients are reasons for further IBD investigations^16^. Diarrhea that is unresponsive to a gluten-free diet and recurrent rectal prolapse are unusual findings in a pediatric celiac disease patient. This persistence of intestinal symptoms and rectal prolapse should be grounds for suspicion of a concomitant IBD. Investigations for IBD in those patients are recommended to prevent advanced manifestations that might include severe malnutrition and edemas like the one we presented here. This also suggests a possible higher rate of undiagnosed IBD-celiac disease patients, especially with the increased risk of IBD in celiac disease patients^11^.

## Conclusions

The overlap between immune-related diseases is a reason to consider further investigations when abnormal findings present in celiac disease patients. This is an important step to avoid severe comorbidities in children with chronic diseases through early diagnosis.

## Ethical approval and consent to participate

Not applicable.

## Consent for publication

Written informed consent was obtained from the patient for publication of this case report and any accompanying images. A copy of the written consent is available for review by the Editor-in-Chief of this journal.

## Sources of funding

Not applicable.

## Author contribution

N.M. is the first author, contributed to drafting, reviewing and editing, corresponding, and bibliography. N.K.T. is a co-first author, contributed to drafting, reviewing and editing. M.A.S. contributed to drafting, reviewing, and editing. J.M. contributed to reviewing, editing, and supervising. All authors read and approved the final manuscript.

## Conflicts of interest disclosure

The authors declare that they have no financial conflict of interest with regard to the content of this report.

## Research registration unique identifying number (UIN)

Not applicable.

## Guarantor

Jaber Mahmoud.

## Data availability statement

Not applicable.

## Provenance and peer review

Not commissioned, externally peer reviewed.

## References

[1] Raiteri A Granito A Giamperoli A . Current guidelines for the management of celiac disease: a systematic review with comparative analysis. World J Gastroenterol 2022;28:154–175. 35125825 10.3748/wjg.v28.i1.154 PMC8793016

[2] Ordonez F Lacaille F Canioni D . Pediatric ulcerative colitis associated with autoimmune diseases: a distinct form of inflammatory bowel disease? Inflamm Bowel Dis 2012;18:1809–1817. 22238154 10.1002/ibd.22864

[3] Aghamohamadi E Asri N Odak A . Gene expression analysis of intestinal IL-8, IL-17 A and IL-10 in patients with celiac and inflammatory bowel diseases. Mol Biol Rep 2022;49:6085–6091. 35526253 10.1007/s11033-022-07397-y

[4] Matsuoka K Kobayashi T Ueno F . Evidence-based clinical practice guidelines for inflammatory bowel disease. J Gastroenterol 2018;53:305–353. 29429045 10.1007/s00535-018-1439-1 PMC5847182

[5] IBD Working Group of the European Society for Paediatric Gastroenterology, Hepatology and Nutrition. Inflammatory bowel disease in children and adolescents: recommendations for diagnosis – the Porto criteria. J Pediatr Gastroenterol Nutr 2005;41:1–7. 15990620 10.1097/01.mpg.0000163736.30261.82

[6] Zempsky WT Rosenstein BJ . The cause of rectal prolapse in children. Am J Dis Child 1988;142:338–339. 3344723 10.1001/archpedi.1988.02150030112034

[7] Yang A Chen Y Scherl E . Inflammatory bowel disease in patients with celiac disease. Inflamm Bowel Dis 2005;11:528–532. 15905699 10.1097/01.mib.0000161308.65951.db

[8] Agha RA Franchi T Sohrabi C . for the SCARE Group. The SCARE 2020 Guideline: Updating Consensus Surgical CAse REport (SCARE) Guidelines. Int J Surg 2020;84:226–230. 33181358 10.1016/j.ijsu.2020.10.034

[9] Ensari A Marsh MN . Diagnosing celiac disease: a critical overview. Turk J Gastroenterol 2019;30:389–397. 31060993 10.5152/tjg.2018.18635 PMC6505646

[10] Volta U Villanacci V . Celiac disease: diagnostic criteria in progress. Cell Mol Immunol 2011;8:96–102. 21278763 10.1038/cmi.2010.64 PMC4003134

[11] Doya LJ Naamah M Karkamaz N . An unusual case of chronic abdominal pain: an association between celiac disease and Crohn’s disease. Oxf Med Case Reports 2021;2021:omab008. 33948181 10.1093/omcr/omab008 PMC8081011

[12] Bramuzzo M Lionetti P Miele E . Phenotype and natural history of children with coexistent inflammatory bowel disease and celiac disease. Inflamm Bowel Dis 2021;27:1881–1888. 33452803 10.1093/ibd/izaa360

[13] Masachs M Casellas F Malagelada JR . Enfermedad inflamatoria intestinal en pacientes celíacos (inflammatory bowel disease in celiac patients) [Spanish]. Rev Esp Enferm Dig 2007;99:446–450. 18020860 10.4321/s1130-01082007000800004

[14] Tse CS Deepak P De La Fuente J . Phenotype and clinical course of inflammatory bowel disease with co-existent celiac disease. J Crohns Colitis 2018;12:973–980. 29741603 10.1093/ecco-jcc/jjy061

[15] Pascual V Dieli-Crimi R López-Palacios N . Inflammatory bowel disease and celiac disease: overlaps and differences. World J Gastroenterol 2014;20:4846–4856. 24803796 10.3748/wjg.v20.i17.4846 PMC4009516

[16] Fasano A Catassi C . Current approaches to diagnosis and treatment of celiac disease: an evolving spectrum. Gastroenterology 2001;120:636–651. 11179241 10.1053/gast.2001.22123

